# Left ventricular function outcome after coronary artery bypass grafting, King Abdullah Medical City (KAMC)- single-center experience

**DOI:** 10.1186/s43044-019-0002-6

**Published:** 2019-08-05

**Authors:** Sheeren Khaled, Ehab Kasem, Ahmed Fadel, Yusuf alzahrani, Khadijah Banjar, Wafa’a Al-Zahrani, Hajar Alsulami, Mazad Ali Allhyani

**Affiliations:** 1 Banha University, Benha, Egypt; 2 King Abdullah Medical City, Muzdallfa Road, Makkah, Saudi Arabia; 3 Zagazig University, Zagazig, Egypt; 4 Monofiya Neurosurgery Hospital, Shibin El Kom, Egypt; 5 Umm Al-Qura University, Al Taif road, Makkah, Saudi Arabia

**Keywords:** CABG, Risk predictors, Left ventricular systolic dysfunction

## Abstract

**Background:**

Coronary artery bypass grafting is known to be associated with better outcome in ischemic heart disease patients with low ejection fraction. We aim to demonstrate the effect of coronary artery bypass grafting (CABG) on left ventricle (LV) systolic function and to identify the predictors that adversely lead to postoperative poor outcome.

**Result:**

This is a cross-sectional prospective study; we included 110 patients with left ventricular ejection fraction (LVEF) < 50% who underwent CABG with a mean age of 56.1 ± 12.2 years old. Those patients were classified into two groups: group I, 76 (69%) patients with LVEF > 35%, and group II, 34 (31%) patients with LVEF < 35%. Our results as regards demographic and clinical data revealed that group II patients had a significantly higher prevalence of diabetes mellitus (DM) and Euro SCORE II compared to group I patients (*p* = 0.05 and < 0.001 respectively); otherwise, all other clinical predictors did not differ between the two studied groups. There was a significant improvement in LVEF post-surgery (*p* = 0.05) in both groups with observed no significant difference recorded for in-hospital mortality rate among patients with different groups. DM, significant diastolic dysfunction, and insertion of IABP are predictors of in-hospital mortality of the patients (*p* = 0.001, 0.03 and < 0.001, respectively)

**Conclusion:**

We concluded that there is a significant improvement of LV systolic function after CABG and hence better survival rate. DM, significant diastolic dysfunction, and perioperative insertion of IABP are predictors of mortality after cardiac surgery. Special care should be provided to such patients to improve their outcome

## Background

Despite improvements in medical therapy and surgical techniques, management of patients with left ventricular (LV) dysfunction and coronary artery disease undergoing cardiac surgery is still challenging [1, 2]. Coronary artery bypass grafting (CABG) has appeared to be superior to medical therapy alone for patients with low ejection fraction (EF), representing a significant clinical improvement and long-term survival [3–7]. For these patients, CABG is associated with higher postoperative morbidity and mortality rates compared with patients with normal EF [5–9].

The studies investigating early postoperative changes have yielded conflicting results. Some have found ventricular improvement within weeks postoperatively [10, 11], while other studies have detected no change [12, 13] or a worsening of ventricular function [14].

Therefore, recognition of the predictors that increase the patients risk for a worse outcome plays a crucial role in the clinical decision-making process [15].

The aim of this study was to assess the effect of cardiac surgery on LV systolic function in patients with abnormal preoperative systolic function and to identify the predictors that may affect the outcome in patients undergoing CABG.

## Methods

It is a cross-sectional, prospective single-center study that included 110 patients who underwent CABG at the cardiac center—King Abdullah Medical City (KAMC)—during 2016 and 2017.

### Inclusion criteria

Patients operated for elective isolated CABG or CABG and mitral valve (MV) repair at KAMC.

### Exclusion criteria

Patients with normal left ventricular ejection fraction (LVEF), severe pulmonary hypertension, cardiogenic shock, atrial fibrillation, combined CABG with other valve intervention, and those who did not have postoperative echocardiography follow-up. Also, patients with very low EF < 20% were excluded as they were rejected by our multidisciplinary team for surgery.

Demographic data (age, gender, body mass index (BMI), etc.), clinical characteristics (diabetes mellitus (DM), hypertension (HTN), renal impairment, old ischemic heart disease (IHD), type of acute coronary syndrome presentation, etc.), and surgical data (ischemic time, bypass time, postoperative intra-aortic balloon pump (IABP), and inotropes) were obtained from medical records.

### Surgical principals

The surgical procedure was performed via median sternotomy with aorto-caval (single/bicaval). Cardioprotection was performed through antegrade mixed with retrograde or direct into the vein after distal anastomosis and topical hypothermia to achieve complete protection of the myocardium. The surgical procedure was done with cardioplegia every 20 min, wean off bypass. Trans-esophageal echocardiography was done for assessment of wall motion and ventricular function after revascularization.

### Echocardiography

All patients had a baseline echocardiogram before and after surgery. Subsequently, study participants were monitored repeatedly throughout the first few months after CABG, in specific regard to their LV function.

Preoperative transthoracic echocardiography (TTE) was collected as well as TTE performed after surgery. Echocardiography was performed with a vivid 7 ultrasound system. Parasternal and apical views were obtained. Ejection fraction is commonly measured by echocardiography, in which the volumes of the heart’s chambers are measured during the cardiac cycle. Ejection fraction can then be obtained by dividing the volume ejected by the heart (stroke volume) by the volume of the filled heart (end-diastolic volume) [16]. Severe LV systolic dysfunction was defined in our cohort study as EF < 35%. Improvement in LVEF was defined as > 5% absolute increase in LVEF in comparison to the preoperative echocardiogram. Consequently, LVEF that decreased by > 5% compared to the preoperative echocardiogram was categorized as worsened. All other postoperative EF measurements within ± 5% of the preoperative values were categorized as unchanged [17]. Other echocardiographic parameters (diastolic function, mitral regurgitation (MR), right atrial (RA) size, right ventricular (RV) size, LV size, left atrial (LA size), right ventricular systolic function (RVSP) and RV function) all were assessed. RV function was evaluated by means of tricuspid annular plane systolic excursion (TAPSE) and tissue Doppler imaging (RV S’).

### Statistical analysis

Statistical analysis was performed by the use of the SPSS software package (SPSS Inc.; Chicago, IL), version 21.0. Data are presented as mean ± SD or as median and range according to the type of distribution of each variable. Chi-square test was used to compare the existence of ventricular dysfunction pre-and postoperatively (logistic DM, HTN, smoking, dyslipidemia, CKD, MI, BNP, LVEF, LV size, TAPSE, RV size). Linear regression analysis was performed. For all analyses, a *p* value < 0.05 was considered significant and not significant if it is > 0.05.

## Results

Out of 264 patients undergoing coronary artery bypass grafting at the cardiac center—King Abdullah Medical City (KAMC)—in the study period, 110 had preoperative LVEF < 50%. Of these, 76 (69%) patients had LVEF > 35% (group I), 34 (31%) had LVEF < 35% (group II), and all were included in the study. Baseline characteristics, comorbidities, type of operation, and intraoperative management of the study population are reported in Table 1.

**Table 1:** Demographics and clinical and operative data

Variable	Number	Percentage
Age	56.1 ± 12.2	–
BMI	27.8 ± 5.9	–
Men	77	70
DM	88	80
HTN	83	75
Obesity	52	47
CKD	18	16
Old IHD	83	75
STEMI presentation	18	16
NEHA II/III	83	75
Standard Euro SCORE	6 (4–8)	–
High BNP	28	25
High troponin	98	89
LV significant systolic dysfunction	34	31
Dilated LV diameter	15	14
Advanced LV diastolic dysfunction	47	43
Dilated RV dimension	9	8
Significant MR	31	28
Preoperative RV dysfunction	11	10
LM disease	15	14
Emergent surgery	4	3.6
CABG alone	87	79
CABG + MVR	23	21
Perioperative IABP	23	20
Perioperative inotropes	6	5
Bypass time	139.41 ± 71.103	–
Cross clamp time	91.09 ± 37.5	–
Incomplete revascularization	37	43
Reoperation for bleeding	6	5.5
Postoperative further deterioration of LVEF	36	33
Postoperative wound infection	20	18
Postoperative AKI	4	4
Postoperative neurological complication	4	4
Mortality	6	5.4

*AKI* acute kidney injury, *BMI* body mass index, *BNP* B-natriuretic peptide, *CABG* coronary artery bypass grafting, *CKD* chronic kidney disease, *DM* diabetes mellitus, *Euro SCORE* European System for Operative Risk Evaluation, *HTN* hypertension, *IABP* intra-aortic balloon pump, *IHD* ischemic heart disease, *LM* left main, *LV* left ventricle, *LVEF* left ventricular ejection fraction, *MR* mitral regurgitation, *MVR* mitral valve replacement, *NEHA* New York Heart Association, *RV* right ventricle, *STEMI* ST-Elevation Myocardial Infarction

Mean age was 56.1 ± 12.2 years, and 70% of patients were male. Mean preoperative LVEF was 29.76 ± 4.868%. Eighty-seven patients (79%) underwent isolated CABG, while 23 patients (21%) underwent combined CABG and mitral valve repair or replacement. Postoperative outcomes are reported in Table 1.

Among our patients, mortality was 5.4% and was consistent with preoperative predictions (mean Euro SCORE).

Preoperative characteristics of the two studied groups were statistically similar except for the significantly higher prevalence of DM and Euro SCORE II in group II. Operative characteristics were statistically similar between both groups (Table 2).

**Table 2:** Comparison of demographic and clinical data between the two groups

Variables	Group I (preoperative LVEF > 35), *N* = 76 (69%)	Group II (preoperative LVEF < 35), *N* = 34 (31%)	*p* value
Age > 65	27 (35.5%)	9 (26.5%)	0.350
DM	57 (75.0%)	31 (91%)	0.05
HTN	57 (75.0%)	26 (76%)	0.117
Smoking	32 (42.1%)	12 (35.3%)	0.500
Dyslipidemia	42 (56.8%)	15 (45.5%)	0.279
CKD	11 (14.5%)	7 (20.6%)	0.423
Obesity	33 (43.4%)	19 (55.9%)	0.226
OLD IHD	56 (73.7%)	27 (79.4%)	0.519
High BNP	19 (55.9%)	9 (75.0%)	0.243
NEHA class II/III	57 (75%)	26 (76%)	0.117
Standard Euro SCORE	6 (4-8)	8 (6–10)	< 0.001
High TROPONIN	64 (84.2%)	34 (100%)	0.014
High Initial SCR	19 (25.0%)	8 (23.5%)	0.868
Dilated LV	8 (10.5%)	7 (20.6%)	0.155
Diastolic dysfunction	26 (34.1%)	21 (61.7%)	0.025
Dilated LA	10 (13.2%)	5 (14.7%)	0.827
Dilated RA	3 (3.9%)	0 (0.0%)	0.240
Dilated RV size	6 (8.0%)	3 (8.8%)	0.885
MR	19 (25.0%)	12 (35.3%)	0.267
TR	5 (6.6%)	4 (11.8%)	2.688
RVD (reduced TAPSE Pre-OP)	4 (5.3%)	7 (20.6%)	0.013
LM disease	11 (14%)	4 (12%)	0.76
Emergent surgery	2 (2.6%)	2 (5.8)	0.08
CABG alone	59 (78%)	28 (82%)	–
CABG + MVR	17 (22%)	6 (18%)	–

*AKI* acute kidney injury, *BMI* body mass index, *BNP* B-natriuretic peptide, *CABG* coronary artery bypass grafting, *CKD* chronic kidney disease, *DM* diabetes mellitus, *Euro SCORE* European System for Operative Risk Evaluation, *HTN* hypertension, *IABP* intra-aortic balloon pump, *IHD* ischemic heart disease, *LA* left atrium, *LM* left main, *LV* left ventricle, *LVEF* left ventricular ejection fraction, *MR* mitral regurgitation, *MVR* mitral valve replacement, *NEHA* New York Heart Association, *RA* right atrium, *RV* right ventricle, *RVD* right ventricular dysfunction, *S CR* serum creatinine, *STEMI* ST-Elevation Myocardial Infarction, *TAPSE* tricuspid annular plan systolic excursion, *TR* tricuspid regurgitation

**Fig. 1:**
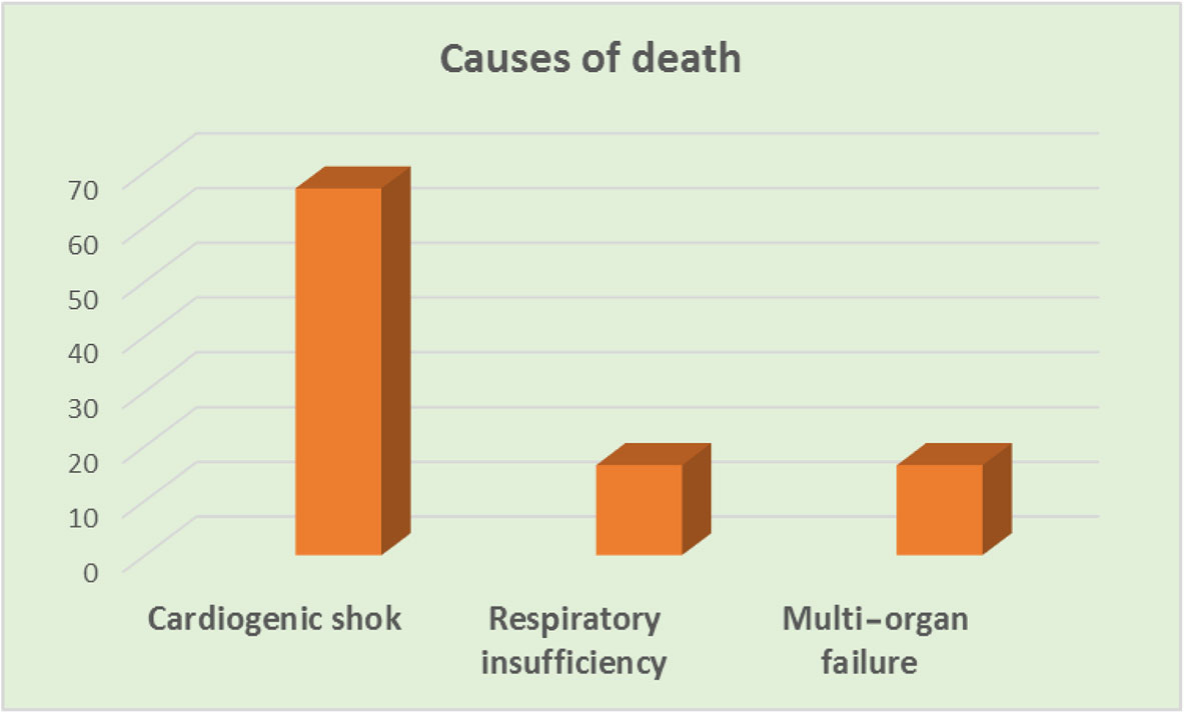
Causes of death among patients

Early 30-day in-hospital mortality was 5.2% in group I and 5.8% in group II, with a statistically insignificant difference. In Table 3, early mortality was observed in one patient (3%) of group II and it was due to cardiogenic shock. The causes of death were cardiogenic shock (3 patients in group I and one patient in group II), multi-organ failure (one patient in group I) and respiratory insufficiency (one patient in group II). There were insignificant differences in postoperative complications, except the significantly higher perioperative insertion of IABP and inotropic support in group II (Table 3 and Fig. 1).

**Table 3:** Operative and postoperative outcome in the studied patients

Variable	Group I	Group II	*p* value
Bypass time	127.24 ± 62.15	141.05 ± 71.31	0.588
Cross clamp time	90.0 ± 40.8	92.3 ± 37.8	0.491
Incomplete revascularization	23 (30%)	14 (41%)	0.21
Perioperative IABP	12 (16%)	11 (32%)	0.009
Perioperative inotropes	1 (1.3%)	5 (14%)	< 0.001
Mortality	4 (5.2%)	2 (5.8%)	0.987
Reoperation for bleeding	5 (6.5%)	1 (3%)	0.35
Postoperative further deterioration of LVEF	27 (35.5%)	9 (26.5%)	0.350
Postoperative wound infection	15 (20%)	5 (15%)	0.67
Postoperative AKI	2 (3%)	2 (5%)	0.56
Neurological complication	3 (4%)	1 (3%)	0.98

*AKI* acute kidney injury, *IABP* intra-aortic balloon pump, *LVEF* left ventricular ejection fraction

### Change of LV function after surgery

Most of our patients had significant improvement of LVEF > 5% in the early postoperative period as the mean LVEF of the whole cohort increased significantly from 29.76 ± 4.868 before surgery to 33.53 ± 9.655 in the first week after surgery. Postoperative improvement in LVEF was also observed in subgroups of patients with a different type of cardiac surgery (Table 4).

**Table 4:** Change in LVEF after CABG

Variable	Preoperative LVEF	Postoperative LVEF	*p* value
Whole cohort	29.76 ± 4.868	33.53 ± 9.655	0.05
Isolated CABG patients	30.00 ± 4.082	35.00 ± 10.408	
CABG + MVR patients	27.00 ± 4.472	31.00 ± 8.944	

*CABG* coronary artery bypass grafting, *LVEF* left ventricular ejection fraction, *MVR* mitral valve replacement

### Predictors of adverse outcome after CABG

DM, significant diastolic dysfunction, and insertion of IABP all were found to be predictors of adverse outcome and mortality in the studied patients (*p* = 0.00, 0.03 and < 0.001 successively). Insertion of IABP was found as an independent predictor of mortality in different groups (Table 5).

**Table 5:** Univariate analysis of perioperative clinical and echocardiography risk predictors associated with in-hospital mortality in the studied patients

Variable	Mortality	Univariate *p* value
All patients	6	
DM	2 (33.3%)	0.001
Diastolic dysfunction II/III	4 (66.7%)	0.03
IABP	4 (66.7%)	< 0.001
Group I	4	
DM	1 (25%)	0.001
IABP	3 (75%)	< 0.001
Group II	2	
Diastolic dysfunction II/III	1 (50%)	0.02
IABP	1 (50%)	0.001

*DM* diabetes mellitus, *IABP* intra-aortic balloon pump

## Discussion

The identification of LVEF after CABG in patients with ischemic cardiomyopathy is considered as a crucial factor to predict the outcome. Improved function and survival following surgical revascularization have been shown in patients with LV dysfunction which is predominantly due to hibernating or stunned myocardium [17]. Our study focused on the effect of CABG on LVEF in patients with baseline impaired systolic function. In this study, a significant improvement in LV systolic function was observed with CABG. This supports the hypothesis that surgical revascularization and restoration of the blood flow to ischemic myocardium preserve already viable and functioning myocardial muscle against later infarction, recruit the hibernating myocardium, and reduce LV remodeling and ischemic burden which all impact LV function recovery.

Because of the lack of routine echocardiographic examination postoperatively, few studies identified the changes in LV systolic function after CABG. Similarly, a recent study was conducted and concluded that successful results of CABG in patients with EF < 50% can be achieved by careful selection of patients and management [18]. Also, another study found that a significant improvement was observed in LV systolic function in patients with preoperative systolic dysfunction [19]. Many factors contributing to the outcome of patients with baseline LV systolic dysfunction after CABG include perioperative care, severity of preoperative LV systolic dysfunction, surgical skills, complete revascularization, type of myocardial protection, cardiac anesthesia management, emergency cardiac facilities, and postoperative intensive care monitoring and management. However, Koene et al. [2] conducted a similar study and concluded that CABG is associated with worse outcome on LV systolic function [20]. A decrease in LV systolic function with CABG surgery could be explained by many factors: significant intraoperative global ischemia which adversely affects the LV function [21] or from myocardial stunning [22] or early postoperative graft failure [23]. In contrast also to our findings, a small study did not find a significant change in LVEF immediately post CABG [24].

Our study demonstrates that in the early mortality, 5.4% is acceptable and in agreement with the current published data [25, 26]. Similarly, Elassy et al. reported higher but insignificant mortality rate in patient with LVEF < 35% (5.6% vs 2.4%) [27]. This is reflecting a marked improvement in the outcome of ischemic cardiomyopathy patients with CABG in highly qualified centers.

### Predictors of adverse outcome with CABG

In this study, we classified our patients into two groups with and without severe LV systolic dysfunction and investigated all the predictors that could lead to poor outcome with CABG.

Type II DM was a significant predictor of adverse outcome of our patients.

Although CABG is considered the best revascularization strategy in diabetic patients, a significantly higher rate of mortality was continually observed in patients with type II DM compared to patients without type II DM [28, 29]. Reasons that have been suggested for that are more aggressive disease with advanced stage of DM in these patients, adverse effects of insulin therapy, hyperinflammatory, and hormonal hyperactivation response [30, 31].

The use of intra- or postoperative IABP was a significant predictor of in-hospital mortality in all patients and when EF was < 50%. Although the use IABP is important to support failing circulation during CABG, it is well known as a risk factor for mortality [32, 33]. It can be explained by that patients who are receiving IABP are already at high risk of increased mortality because of unstable hemodynamic status and its complications (stroke, paraplegia, limb ischemia, infection, and hemolysis) that all have an impact on mortality after CAGB [34, 35].

We also demonstrated advanced diastolic dysfunction as a predictor of adverse outcome and mortality in patients with LV dysfunction. Diastolic dysfunction has been reported to be an independent risk predictor of postoperative heart failure, atrial fibrillation, and cardiac death in different studies [36, 37].

Finally, a limited number of patients are included due to the nature of a single center.

Some patients also did not have echocardiography follow-up, hence excluded from our study. Follow-up echocardiography was done few months only post CABG, and thus, our results could not be correlated with long-term outcome after surgery. Like other studies evaluating adverse outcome after CABG in patients with reduced LVEF, data regarding patency of grafts were not applicable in our study. The results of this study are encouraging, and it needs corroboration in multicenter larger population with longer follow-up.

## Conclusion

This study confirmed that there is a remarkable improvement of LV systolic function after coronary artery bypass grafting and that reflects the high benefit of CABG in patients with reduced EF. Diabetes mellitus, high Euro SCORE, advanced diastolic dysfunction, and insertion of IABP were significant predictors of adverse outcome. So, identification of patients with those risk predictors could provide complementary prognostic information and help to maximize the care, monitoring, and close follow-up to improve their expected poor outcome. More investigation is required for similar data in other tertiary centers to provide multicenter results and hence generalize our conclusion.

## Data Availability

The data that support the findings of this study are available on reasonable request from the corresponding author but are not publicly available due to privacy.

## Abbreviations

AKI: Acute kidney injury
BMI: Body mass index
BNP: B-natriuretic peptide
CABG: Coronary artery bypass grafting
CKD: Chronic kidney disease
DM: Diabetes mellitus
Euro SCORE: European System for Operative Risk Evaluation
HTN: Hypertension
IABP: Intra-aortic balloon pump
IHD: Ischemic heart disease
LA: Left atrium
LM: Left main
LV: Left ventricle
LVEF: Left ventricular ejection fraction
MR: Mitral regurgitation
MVR: Mitral valve replacement
NEHA: New York Heart Association
RA: Right atrium
RV: Right ventricle
S CR: Serum creatinine
STEMI: ST-Elevation Myocardial Infarction
TAPSE: Tricuspid annular plan systolic excursion
TR: Tricuspid regurgitation
